# Oxidative Stress and Chronic Myeloid Leukemia: A Balance between ROS-Mediated Pro- and Anti-Apoptotic Effects of Tyrosine Kinase Inhibitors

**DOI:** 10.3390/antiox13040461

**Published:** 2024-04-13

**Authors:** Alessandro Allegra, Giuseppe Mirabile, Santino Caserta, Fabio Stagno, Sabina Russo, Giovanni Pioggia, Sebastiano Gangemi

**Affiliations:** 1 Division of Hematology, Department of Human Pathology in Adulthood and Childhood ‘Gaetano Barresi’, University of Messina, 98125 Messina, Italy; giuseppe.mirabile@polime.it (G.M.); santino.caserta@polime.it (S.C.); fabio.stagno@unime.it (F.S.); sabina.russo@polime.it (S.R.); 2 Institute for Biomedical Research and Innovation (IRIB), National Research Council of Italy (CNR), 98164 Messina, Italy; giovanni.pioggia@irib.cnr.it; 3 Allergy and Clinical Immunology Unit, Department of Clinical and Experimental Medicine, University of Messina, 98100 Messina, Italy; gangemis@unime.it

**Keywords:** chronic myeloid leukemia, oxidative stress, reactive oxygen species, apoptosis, chemoresistance, BCR-ABL1, tyrosine kinase inhibitors, fenretinide, nanoparticles, RNA interference

## Abstract

The balanced reciprocal translocation t (9; 22) (q34; q11) and the BCR-ABL fusion gene, which produce p210 bcr-abl protein production with high tyrosine kinase activity, are characteristics of chronic myeloid leukemia, a myeloproliferative neoplasm. This aberrant protein affects several signaling pathways connected to both apoptosis and cell proliferation. It has been demonstrated that tyrosine kinase inhibitor treatment in chronic myeloid leukemia acts by inducing oxidative stress and, depending on its level, can activate signaling pathways responsible for either apoptosis or survival in leukemic cells. Additionally, oxidative stress and reactive oxygen species generation also mediate apoptosis through genomic activation. Furthermore, it was shown that oxidative stress has a role in both BCR-ABL-independent and BCR-ABL-dependent resistance pathways to tyrosine kinases, while patients with chronic myeloid leukemia were found to have a significantly reduced antioxidant level. The ideal environment for tyrosine kinase inhibitor therapy is produced by a favorable oxidative status. We discuss the latest studies that aim to manipulate the redox system to alter the apoptosis of cancerous cells.

## 1. Introduction

### 1.1. General Consideration on Chronic Myeloid Leukemia and Oxidative Stress

Chronic myeloid leukemia (CML) is defined by the unchecked development of myeloid cells at various stages of maturity. The three clinical phases of CML that are traditionally recognized are the blastic, rapid, and chronic stages [1,2]. The Philadelphia (Ph)-chromosome, or translocation t (9; 22), has been recognized as the hallmark of CML [3], and the BCR-ABL1 fusion gene has subsequently been identified as the key participant in the pathophysiology of CML. A 210 KD chimeric protein with constitutively active tyrosine kinase activity is encoded by BCR-ABL1, and in neoplastic cells it activates multiple downstream signaling pathways [4]. Specifically, this oncoprotein’s expression causes changed adherence to extracellular matrix and stromal cells, which promotes survival and inhibits apoptosis [3].

Clinical results have been markedly improved using BCR-ABL tyrosine kinase inhibitors (TKIs) such as dasatinib, nilotinib, and imatinib. Even in patients who have remission, primitive leukemia CD34+ stem/progenitor cells are still present, indicating that TKI therapy is not enough to cure CML [5,6].

Because of all of this, it is now critical to identify new pathogenetic events and potential treatment targets. A state of physiological balance between intracellular reactive oxygen species (ROS), reactive nitrogen species (RNS), thiol-containing chemicals, and the antioxidants that regulate their removal is known as cellular redox homeostasis. As byproducts of oxygen metabolism, endogenous ROS are mostly formed in the mitochondria. The mitochondrial oxidative phosphorylation (OXPHOS) system consists of four enzyme complexes in the respiratory chain that transport electrons from the oxidation of lipids and carbohydrates to molecular oxygen (O_2_); the energy from this process powers the creation of ATP by a fifth enzyme complex. Complex IV of the mitochondrial electron transport chain (ETC) catalyzes the process that converts O_2_ into H_2_O, which is the electron acceptor. Electron transport to O_2_ may take place in complexes I or III due to partial process efficiency, which can lead to the production of ROS that have the ability to damage lipids, nucleic acids, and proteins in cells. When cells are subjected to medications that alter electron transport in the ETC, the resulting ROS results in cell malfunction or death [7]. Furthermore, external environmental stimuli such as heavy metals, xenobiotics, ultraviolet (UV) and ionizing radiations, certain compounds, and pollutants can also produce ROS. Finally, NADPH oxidase is an enzyme whose function is to produce large amounts of ROS in a respiratory burst characterized by consumption of O_2_ and production of superoxide as well as secondary ROS products. The catalytic subunit, gp91phox—now referred to as Nox2—is a flavocytochrome that utilizes electrons from cytosolic NADPH to reduce molecular oxygen to form superoxide. The enzyme, present in neutrophils and other phagocytic cells, is dormant in circulating neutrophils, but becomes activated upon exposure to microbes, microbial products, or inflammatory mediators, producing large amounts of ROS as part of microbicidal mechanisms. There, when activated, Nox2 generates high concentrations of ROS [8].

At the physiological level, ROS control cell division, proliferation, and death through their role as second messengers in intracellular Ca^2+^ signaling pathways [9]. But long-term increases in free radicals damage proteins, lipids, and deoxyribonucleic acid (DNA) in addition to starting ROS signaling cascades that intensify cellular oxidative stress (OS). In addition, an iron-dependent rise in ROS levels triggers autophagy activation, p53-dependent cell death, necrosis induction, ferroptosis, and lipid peroxidation-mediated cell death [10].

Evidence has demonstrated OS’s role in both the pathophysiology and response to specific therapies of hematological disorders [11,12,13,14,15,16,17,18,19]. Several aspects of CML have been linked to OS, including dominance of one or more malignant clones or subclones, DNA alteration and defective DNA repair, antiapoptotic effect of p210 protein, and genomic instability in hematopoietic stem cells associated with bcr-abl oncoprotein [20,21,22].

As with other malignancies, CML is typified by genetic heterogeneity that is triggered by the BCR-ABL1 kinase, at least partially. The kinase can increase ROS generation, which can lead to cellular redox imbalance and DNA damage [23]. Increased vulnerability to exogenous oxidative stress brought on by external variables may result from such endogenous OS. BCR-ABL1-related pathways can misrepair DNA damages caused by ROS [24,25,26]. The primary determinant of genomic instability is the cellular DNA damage response (DDR), where DNA repair is a critical component. In numerous cell types, BCR-ABL1 modulates DNA repair [27,28,29,30].

Additionally, an earlier work revealed that BCR-ABL1-induced genomic instability may be linked to imatinib resistance as well as the cancer phenotype of BCR-ABL+ cells [31]. Exogenous ROS can modify the structure of BCR-ABL1 due to its redox-sensitive cysteine residues. This can disrupt the protein’s interaction with small molecules and lead to the development of imatinib resistance [32].

Point mutations typically occur when heightened oxidative DNA damage brought on by ROS is not repaired. Studies demonstrated that not only imatinib-naive, but also imatinib-treated LSCs and/or LPCs from CML-Chronic phase (CP) patients and CML-CP–like BCR-ABL1 transgenic mice contained 2–6 times more ROS and oxidized bases in comparison with their normal counterparts, highlighting the potential role of ROS-induced oxidative DNA damage in the accumulation of point mutations in CML-CP cells. Antioxidants like vitamin E also decreased ROS and the build-up of BRC-ABL1 kinase mutations that are resistant to TKIs in vitro.

This may be a very important point to note because antioxidants like vitamin E can help prevent TKI resistance, especially resistance caused by mutations in the BCR-ABL1 kinase. Furthermore, as TKI-resistant mutations are produced by large levels of ROS accumulated by imatinib-treated CML-CP leukemia stem cells (LSCs) and leukemia progenitor cells (LPCs), antioxidant therapy may be used in conjunction with TKIs to increase or prolong the therapeutic benefits of ABL1 kinase inhibitors. The fact that the antioxidants vitamin E and N-acetyl cysteine decreased the proportion of resistant clones that emerged in vitro from BCR-ABL1-positive cells treated with imatinib lends credence to this theory [31,33].

As we will see below, evidence has also been presented that OS participates in TKI resistance mechanisms as well as BCR-ABL-independent resistance mechanisms [34].

### 1.2. Chronic Myeloid Leukemia, Oxidative Stress, and Apoptosis

OS’s impact on apoptosis is a distinct and significant factor in the etiology and development of CML. The start and transmission of death signals, the activation of death programs, and the elimination of apoptotic cells are the distinct processes that make up apoptosis. The inhibitor of apoptosis family (cellular inhibitor of apoptosis protein-1 or cIAP1, Livin, X-Linked Inhibitor Of Apoptosis or XIAP, NLR Family Apoptosis Inhibitory Protein or NAIP, Baculoviral IAP repeat-containing protein3 or C-IAP2, and Survivin) inhibits the activity of various caspases to prevent apoptosis, while the extrinsic death receptor pathway and the intrinsic mitochondrial pathway are the two main apoptotic pathways that activate the caspase protease family [35]. The interaction of ligands and death receptors results in the extrinsic route. When the death-inducing signaling complex is assembled and caspase-8 and caspase-3 are activated, the tumor necrosis factor receptor (TNFR) family of death receptors and associated ligands are stimulated. Damage to DNA, malfunctioning mitochondria, and environmental stress all contribute to the intrinsic route. Damage to the mitochondria alters the permeability of the outer membrane of the mitochondria, causing cytochrome c to be released, which interacts with the apoptotic protease-activating factor-1 to set off the caspase cascade response [36]. Furthermore, the peptidase family C2 (calpain family) contains calcium-dependent cysteine proteases called calapains (CAPNs), which are crucial for pro-death following Ca^2+^ overload. Specifically, CAPNs trigger the release of cytochrome c via cleaving caspase 3, apoptosis-inducing factor (AIF), and Bid. By raising intracellular Ca^2+^ levels, ROS can indirectly start CAPN activation. Alternatively, they can start it directly by causing the proteases to undergo oxidative alterations. Furthermore, the susceptibility of many proteins to CAPN-induced cleavage can be impacted by changes mediated by ROS. When nicotinamide adenine dinucleotide phosphate (NADPH) oxidase is inactivated, ε-CAPN activity is attenuated, and pulmonary microvascular endothelial cell death is reduced. Additionally, it was demonstrated that giving an antioxidant called Trolox stopped caspase 3 and µ-CAPN from activating, suggesting that oxidative stress is necessary for the system to activate [37].

Finally, through genetic activation, OS and ROS production also mediate apoptosis [38].

Regarding the genomic instability brought on by oxidative stress, ROS directly damage DNA, resulting in many types of DNA injury, including strand breaks. These damage events impact the expression levels of important genes, including proto-oncogenes, oncogenes, and genes involved to DNA damage repair, and they also encourage the growth of leukemic cells. Furthermore, it has been clarified that ROS triggers the SOS response and mutagenic break repair by breaking bases in DNA, which pauses the replisome and permits the crucial transition from high fidelity to error-prone DNA polymerases, which in turn causes more carcinogenic mutation. It has been also demonstrated that ROS can influence cysteine residue oxidation, which activates the most prevalent oncogenic switch genes in human malignancies, and hence influence tumorigenesis and transformation [39].

The process of apoptotic cell death involves several genes. When DNA damage or cellular stress occurs, the P53 protein causes cell cycle arrest to give time for damage repair or self-mediated apoptosis [40].

### 1.3. The Bcl-2 Family Genes Are Involved in Apoptosis Induction Mechanisms

**Figure 1:**
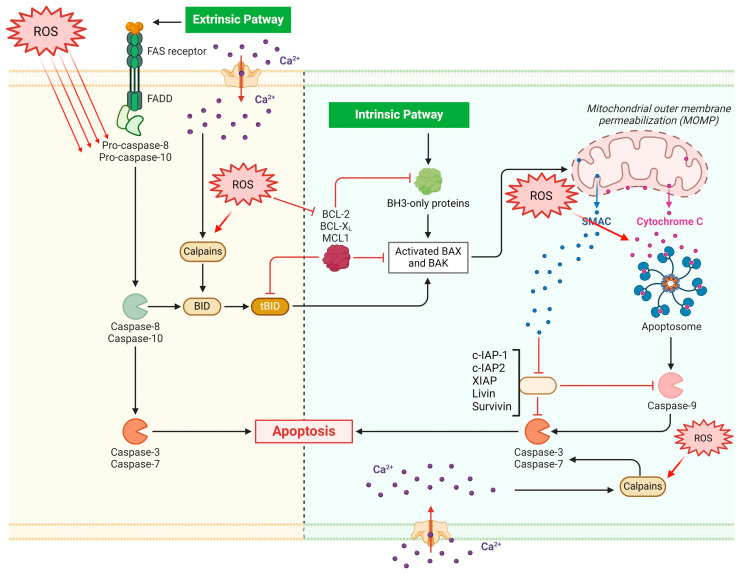
ROS are directly implicated in apoptotic dynamics since they may encourage genomic instability and may facilitate the apoptosis of leukemic cells. Both intrinsic and extrinsic pathways are involved in apoptosis via the effects on caspases, Calpains, and aptosome. While the extrinsic pathway is dependent on cell-surface death receptors such as Fas (First apoptosis signal), the intrinsic pathway is initiated within mitochondria. BID BH3 interacting-domain death agonist; Mcl-1 myeloid leukemia cell differentiation protein; IAP1 inhibitor of apoptosis 1; BH3 Bcl-2 homology domain 3.

The Bcl-2 family, which comprises Bcl-2 and Bax, plays a role in regulating apoptosis. Specifically, pro-apoptotic Bax induces apoptosis, while antiapoptotic Bcl-2 family members such as Bcl-2 prevent or postpone cell death [41]. The determination of a cell’s fate in response to an apoptotic stimulation is based on the ratio of Bax/Bcl-2. Apoptosis induction results from a loss in cellular tolerance to apoptotic stimuli caused by an increase in the Bax/Bcl-2 ratio [42]. Furthermore, ROS and RNS regulate Bcl-2 expression levels, thereby impacting its function on the apoptosis machinery [42] (Figure 1). However, an important role is also played by antioxidant effects of apoptosis inhibitors, including Bcl-xL [43].

As a result, OS may have two distinct roles in the development of CML: first, as previously mentioned, it may encourage genomic instability and hasten the disease’s advancement to later stages linked to resistance to tyrosine kinase inhibitors; second, it may facilitate the apoptosis of leukemic cells.

Reactive oxygen species levels in leukemic cells appear to be directly related to the delicate balance between their pro- and anti-apoptotic actions [44].

The oxidation of biomolecules, comprising either the hydrogen abstraction by superoxide anion and hydroxyl radicals or the cycloaddition of singlet oxygen, initiate a cascade of oxidative reactions that lead to the formation of electronically excited species, such as triplet-excited state and singlet oxygen. Thus, there are two main types of ROS: oxygen radicals are produced by electron transfer (e.g., superoxide anion O_2_^•−^, hydroxyl radical HO^•^, hydroperoxyl radical HOO^•^), while singlet oxygen (^1^O_2_) is obtained by energy transfer [45].

Elevated levels of ROS within cells offer a direct means of causing necrotic cell death, but a slight rise in ROS, such as H_2_O_2_ or O_2_, can function as a prooxidant condition and shield cells from killing themselves [46]. On the other hand, a decreased state and an H_2_O_2_/O_2_ ratio that promotes lowering of the intracellular milieu make cells more susceptible to apoptotic stimuli, which may result in spontaneous apoptosis [47]. Furthermore, ROS have been assigned a role as signaling molecules in immunity, based in part on the discovery that lymphocyte effector cells, including cytotoxic T and natural killer cells, experience cell death akin to apoptosis when coming into contact with myeloid cells that produce ROS [48].

Finally, ROS can modify apoptosis with different mechanisms and with very different effects. In fact, ROS generated as a result of the small GTPase Rac-1’s participation with integrin engagement play a crucial function in transducing a pro-survival signal that guarantees cells escape anoikis. Specifically, a study showed that ROS were in charge of the redox-mediated activation of Src, which ligand-independently trans-phosphorylates the epidermal growth factor receptor (EGFR). The extracellular signal-regulated protein kinase and Akt downstream signaling pathways are both activated by the redox-dependent phosphorylation of EGFR, which leads to the degradation of the pro-apoptotic protein Bim. These, in turn, demonstrate the mechanism responsible for the adhesion-dependent antiapoptotic impact, emphasizing the crucial role that ROS-mediated Src regulation plays in maintaining anoikis defense [49].

### 1.4. What the Role of ROS in the Apoptosis of Cancer Cells?

Numerous studies have been conducted on the role of ROS in apoptosis [50]. It is well known that the level of oxidative stress in cancer cells was higher than in matching healthy cells, and that antioxidant molecules like glutathione and SOD regulate the rise in intracellular ROS [51]. Researchers frequently employed the manipulation of the changed state, with both oxidants and pro-oxidants, to kill cancer cells [52].

Actually, when cellular antioxidant function is inhibited, ROS levels rise. These elevated ROS levels can then activate signaling pathways, potentially rendering cancer cells more vulnerable to toxic assaults [53,54]. Numerous anticancer medications, including dexamethasone and anti-IgM antibodies, cause apoptosis by increasing ROS and H_2_O_2_ buildup, but many apoptosis inhibitors also have antioxidant qualities [55]. According to recent research, hydroxychavicol, a strong xanthine oxidase inhibitor that can be synthesized or purified from Piper betle leaves, can activate JN-kinase ROS-dependent apoptosis in K562 and CML cells and reverse drug resistance brought on by imatinib [56]. A prior study’s findings show that when administered in vitro, LY294002, a PI3K tyrosine kinase inhibitor, and a Src kinase inhibitor (PP1) work in concert with imatinib to cause autophagy and apoptosis in CML and K562 cells. This mechanism is associated with endoplasmic reticulum stress [57,58]. In fact, in eukaryotic cells, the endoplasmic reticulum (ER) is a vital organelle. Normally, the important location for protein folding is the ER. The unfolded protein response (UPR) is responsible for monitoring and maintaining the quality control of protein folding. A stress response known as ERS is brought on by an excessive stack of unfolded proteins in the ER lumen because of incorrect protein folding and processing when the organism is exposed to harmful external stimuli. In response to the unfavorable external circumstance, UPR is simultaneously engaged as a homeostatic network to promote protein folding, reduce ERS, and restore ER equilibrium. However, in circumstances of severe and protracted ERS, UPR fails to return the ER to equilibrium, which results in cellular death. Small-scale ROS production is possible in a number of membrane organelles, including the ER and mitochondria, under healthy conditions. On the other hand, oxidative stress develops when the equilibrium between producing and scavenging free radicals derived from oxygen is upset. Numerous signaling pathways, including PI3K/Akt, MAPK, NF-κB, p53, and nuclear factor erythroid 2-related factor 2 (Nrf2)/Keap1, are activated by this process and contribute to activities relevant to cell survival or death [59].

Then, the various activities of various tyrosine kinase inhibitors may be related to the impact of OS on the induction of apoptotic events. In relation to the interaction between OS and TKI therapy, it is known that low dose imatinib treatment enhances antioxidant defense, whereas dasatinib treatment decreases antioxidant status and increases ROS in different tissues [60]. Lower levels of imatinib caused an augment of glutathione concentration following its reduction at higher imatinib levels, suggesting diminishing oxidative stress protections. Concurrent to a glutathione reduction, an augment of malondialdehyde and protein carbonyls concentration was reported, suggesting oxidative impairment of proteins and lipids [58]. Dasatinib effects on endothelial cells are due to a ROS-dependent mechanism. This was demonstrated in vitro and in vivo employing a treatment with the antioxidant substance, *N*-acetylcysteine. Various cell types were also used to demonstrate this effect.

**Figure 2:**
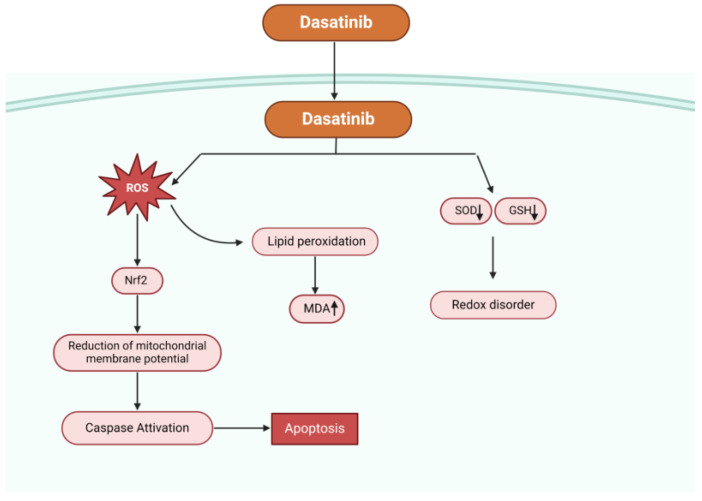
Possible mechanisms of dasatinib-mediated apoptosis. Dasatinib increases the concentration of ROS, reduces the intracellular glutathione content (GSH), reduces the activity of superoxide dismutase (SOD), causes lipid peroxidation producing malondialdehyde (MDA), decreases the mitochondrial membrane potential, and activates nuclear factor erythroid 2-related factor 2 (Nrf2) and mitogen-activated protein kinases (MAPK) related to oxidative stress.

In an experiment, dasatinib significantly raised the amount of ROS in hepatocytes, decreased the amount of intracellular glutathione (GSH), inhibited the activity of superoxide dismutase (SOD), produced malondialdehyde (MDA) (a byproduct of lipid peroxidation), lowered the potential of the mitochondrial membrane, and activated mitogen-activated protein kinases and nuclear factor erythroid 2-related factor 2 (Nrf2), which are linked to oxidative stress and survival (Figure 2). These findings demonstrated the critical role oxidative stress plays in dasatinib-mediated cytotoxicity. Typical antioxidants such n-acetyl-cysteine can reduce oxidative stress, scavenge free radicals, and shield hepatocytes from damage caused by dasatinib [61].

Moreover, the authors examined the oxidative state of CML patients with significant molecular responses (MMR) and found that, in comparison to patients who received MMR after second generation TKI treatment, MMR-I patients had higher mean total antioxidant capacity (TAC) values and lower mean ROS values. This aspect implies that the best conditions for the therapeutic effect of first-generation TKIs are created by a favorable oxidative status (quantitatively reduced ROS and effective antioxidant systems), whereas the use of second-generation TKIs is necessary to obtain MMR in an oxidative status with high ROS concentrations and inadequate antioxidant defense [62,63,64].

Conversely, research indicates that dasatinib and nilotinib’s apoptotic activity produces ROS and oxidative chemicals. Apoptotic effect of dasatinib and nilotinib is probably due to an augment of the production of oligonucleosomes by causing extreme ROS production [65]. Bazi et al. have established that TKI therapy for CML works by inducing OS. OS can reduce transcriptional function of UPR-related survival transcription factor, spliced X-box binding protein 1 (sXbp1) which is involved by suppressed expression of Glucose-regulated protein 94 (Grp94) in combinational states. This OS can be used to initiate both apoptotic and survival processes inside leukemic cells, depending on the extent of induction, which can be either favorable or unfavorable [66].

In peripheral blood leukocytes from recently diagnosed CML patients (CML-PBM), researchers assessed the in vivo effects of dasatinib, nilotinib, and imatinib on intracellular calcium concentration, oxidative stress, and apoptosis [67].

The finding showed that treatment with dasatinib and nilotinib, which inhibit the activities of lithium, an inositol 1,4,5-triphosphate receptor inhibitor, and thapsigargin, a sarcoplasmic/endoplasmic reticulum Ca2ξ-ATPase inhibitor, had a higher modulating potential than imatinib on intracellular calcium concentration. Furthermore, these findings showed that by affecting both oxidative stress and intracellular calcium signaling, dasatinib and nilotinib enhanced apoptosis more than imatinib. Acquiring data on the values of calcium channel receptors and OS stress may enable the hematologist to better control and manage CML disease.

These results were validated by additional research. A higher degree of OS was observed in CML patients treated with second-generation TKIs in comparison to those treated with first-generation TKIs in a distinct investigation. It was believed that patients who exhibit resistance to first-generation TKIs and who have a high degree of OS should start second-generation TKI therapy [68].

Ammar et al. showed that oxidative marker concentrations in individuals with imatinib resistance are considerably greater than in patients without imatinib resistance [69]. When resistant CML patients were compared to non-resistant ones, the resistant patients had greater MDA levels as well as CAT and SOD activity. Activity on GPx decreased. Additionally, IM-resistant patients had higher mean ratios of MDA/GSH, MDA/GPx, and SOD/(GPx + CAT) than non-resistant patients. Compared to IM non-resistant CML patients, the mean ratio of GPx/GSH was lower in IM-resistant patients. Authors observed a negative association between MDA level and the ratio SOD/(CAT + GPx) in IM-resistant individuals and a positive correlation for SOD and (CAT + GPx) activities, as well as between GSH level and GPx activity. It appears that O_2_^•−^, and in particular H_2_O_2_, plays a significant role in the development of resistance to IM therapy, which may aid in the emergence and/or advancement of more severe diseases [69].

The existence of cellular clones resistant to imatinib is linked to the overproduction of ROS, as confirmed by Nieborowska-Skorska [32]. But one must remember that some research implicates the OS in causing genetic changes that result in TKI resistance [70].

The TAC value is substantially lower in patients who are resistant to dasatinib than in patients who received MMR, which supports the view of other researchers that antineoplastic treatments would work better in a redox-balanced state if mitochondrial ROS production was reduced [71,72].

## 2. Induction of Apoptosis via Modification of Oxidative Stress

CML cell death may increase with the potential use of drugs or treatments that alter the redox state. We shall assess the most current studies that aim to alter neoplastic cells’ apoptosis by modifying the redox system in the upcoming sections.

### Natural Products

Because of their anti-inflammatory, pro-apoptotic, antioxidant, and anticarcinogenic properties, polyphenols derived from various sources have therapeutic benefits in a variety of pathophysiological situations [73,74,75].

Curcumin, one of the polyphenols, interacts with most targets that are modified by FDA-approved medications and possesses chemotherapeutic qualities via several pathways [76,77,78].

Although curcumin is a promising option for an anticancer medication, its therapeutic efficacy is restricted by its low solubility and limited bioavailability [79]. It has been hypothesized that combining with other polyphenols that are highly soluble and have an enhancement effect on phytochemical bioavailability will help get around these restrictions.

A study showed that by altering the oxidant/antioxidant status, mitochondrial membrane potential, and inducing intrinsic apoptosis in K562 cells, the synergistic dose of quercetin and curcumin triggered apoptosis and reduced growth [80]. Another study looked at how the combination of quercetin and curcumin affected the expression of several signaling pathway genes in K562 and HMCL cells. In this instance, the genes under investigation were connected to signaling pathways for oxidative stress [81]. Different natural chemicals have been utilized in other investigations. The flavan brosimine B present in the bark of *Brosimum acutifolium* exhibits anti-inflammatory and anti-rheumatic properties [82,83]. Additionally, it has been shown to have cytotoxic action on vincristine-sensitive and -resistant cells in a lymphoma cell line. Numerous plants, fruits, and vegetables contain betulinic acid (BA), a pentacyclic triterpene with a number of medicinal uses, including anti-inflammatory, antiviral, antibacterial, antimalarial, immunomodulatory, antidepressant, and anticancer properties [84,85,86]. Several tumor lines have reported the anticancer efficacy of BA [87,88]. Its main mode of action is the induction of cell death by many mechanisms, such as apoptosis, kinase protein regulation, autophagy, and suppression of DNA topoisomerases.

In K562 sensitive to imatinib CML cell lines and K562-R resistant to imatinib CML cell lines, a study examined the cytotoxic activity and potential mode of action of a hybrid compound of BA and brosimine B. It also assessed lower dosages of imatinib in combination with the hybrid drug [89]. The compound’s effects on oxidative stress, autophagy, cell cycle, and apoptosis were studied, as well as the consequences of combining it with imatinib.

In K562 and K562R cells, the chemical was cytotoxic, and when it was combined with imatinib, a synergistic effect was seen. The caspase 3 and 9 intrinsic pathway induced apoptosis, and cell cycle analysis revealed arrest at G0/G1. Furthermore, the hybrid chemical upregulated Beclin-1 and LC3II mRNA levels, which in turn promoted autophagy and enhanced the generation of reactive oxygen species. The results point to the hybrid compound’s potential as a novel anticancer treatment for CML since it kills both K562 and K562-R cell lines.

The cytotoxic potential of hybrid compounds has been assessed in a number of further investigations. For their epidermal growth factor receptor (EGFR) tyrosine kinase activity, triazole-substituted quinazoline derivatives have been produced and have shown modest activity in tumor cell lines (HCT116, MCF-7, and PC-3) [90]. When hybridized with a synthetic version of artemisinin, BA shows tumor cell activity in various tumor cells [91]. To increase BCR/ABL inhibitory activity in CML cells, hybrid compounds including portions of the structures of imatinib, nilotinib, and dasatinib have been created [92].

Through OS regulation, other drugs can cause CML cells to undergo apoptosis. N-4-hydroxyphenyl-retinamide (4-HPR) or fenretinide is a synthetic retinoid that produces dihydroceramide, which is what causes cytotoxicity. Fenretinide can cause cancer cells to undergo apoptosis by OS, according to a number of studies [93,94]. According to a paper, results point to a variety of processes, perhaps related to the heterogeneity of CD34+ cells, which underlie the apoptosis induced in CD34+ CML cells. However, the subsequent redox signaling processes that cause apoptosis seem more conclusive, as evidenced by the manifestation of normal ER stress and UPR.

On the other hand, OS responses in a recent investigation of fenretinide on CD34+ AML cells [95] were not as noticeable as those in CD34+ CML treated with fenretinide. AML stem/progenitor cells were less carcinogenic and more like hematopoietic stem/progenitor cells than CML stem/progenitor cells, which is one possible cause. Analysis of the highly regulated genes in this context suggests that transcription factors, such as nuclear factor erythroid-derived 2 (NF-E2), TCF11/MafG (NRF1), and AP-1, are significant regulators of genes participating in the stress-responsive c-Jun N-terminal kinase (JNK) pathway.

On the other hand, transcription factors’ responses to fenretinide therapy in cancer cells can vary and rely on the kind of cell.

Leukemic NB4 cells, for example, appear to undergo fenretinide-induced apoptosis as a normal oxidative stress-mediated mechanism, involving several stress-responsive processes such as ER stress and UPR, and are transcriptionally regulated by NRF2 and HSF1. As predicted, ROS increased rapidly following treatment, quadrupling relative to basal levels in just six hours. However, ROS then surprisingly dropped off over the next six hours to levels roughly equal to twice that of untreated cells’ basal levels. This evidence implies that NB4 cells treated with fenretinide have redox signaling involved. Intracellular ROS quickly accumulate as a result of fenretinide activation, and this may trigger cellular defense systems to lower ROS concentrations. Furthermore, fenretinide-induced apoptosis is likely dependent on moderate intracellular ROS levels [96]. Furthermore, there may be some variations in the stress-activated signaling pathways in cancer cells treated with fenretinide. Ceramide signaling is crucial for oxidative stress-mediated apoptosis in most cancer cell types. However, in three leukemic cell lines treated with fenretinide (NB4, U937, and HL-60), this signaling is only essential for apoptosis in HL-60 [97].

Fenretinide and fenretinide plus imatinib significantly decreased the number and size of total colonies created from CD34+ CML cells, as demonstrated by colony-forming cell tests performed on clinical specimens. Specifically, colonies originating from pluripotent/multipotent and erythroid progenitors exhibited heightened sensitivity to fenretinide or fenretinide in combination with imatinib. In line with this, fenretinide seemed to cause apoptosis in CD34+ CML cells, especially in the case of CD34+CD38- cells. Results from cell quiescent assays, such as the Ki-67 negative test, provided more proof that fenretinide significantly reduced the number of nonproliferative CD34+ CML cells. Furthermore, transcriptome and molecular data demonstrated the complexity of the mechanisms underlying the apoptosis in CD34+ CML cells, which involved several OS response events.

When combined with imatinib, fenretinide and imatinib may provide a more complex approach to treating chronic myeloid leukemia (CML). While fenretinide primarily targets CML stem/progenitor cells via the oxidative/endoplasmic reticulum stress-mediated pathway, imatinib primarily targets leukemic blast cells through the intrinsic pathway of apoptosis [97].

Additionally, compounds with imidazo[1,2-a] pyridines (IPs) in their core structure have found extensive application in drug development and medicinal chemistry. This is because there is a correlation between these chemicals and intriguing therapeutic features, such as anti-inflammatory and antineoplastic activities [98,99,100,101]. In addition, Ips have a reputation for being strong P3IK/mTOR inhibitors that induce cell-cycle arrest and death with potential kinase selectivity [102].

Conversely, a variety of biological processes, such as OS, ROS overproduction, DNA damage, and mitochondrial dysfunction, are modulated by organoselenium compounds [103].

Due to their pro-oxidant effects on tumor cells and biocompatibility, organoselenium compounds show intriguing anti-cancer capabilities [104]. Human leukemia cells Kasumi, KG-1, K562, and Jurkat were subjected to redox screening. For experiments measuring cytotoxicity, cell proliferation, cell senescence, and oxidative stress, the IP derivative and the leukemic cell with the highest response were chosen. Predictive toxicity investigation revealed no mutagenic, carcinogenic, or irritable effects, but rather a potential impact on the reproductive system. In contrast to K562 cells, MRK-107 was the chemical with the best redox profile. In K562 and monocyte cells, MRK-107 did not cause cell death.

After 48 and 72 h, however, this chemical was able to increase cell senescence and inhibit cell growth. Moreover, after 72 h, MRK-107 caused oxidative stress in K562 cells, which decreased the levels of reduced glutathione (GSH) and increased lipid peroxidation. This work addressed MRK-107 as a prospective anticancer medication against CML by demonstrating that oxidative stress-induced senescence is a plausible mechanism of action [105]. The molecular hybridization of these two chemical structures yields some intriguing pharmacological features, which are relevant to both the biological significance of organochalcogen compounds and the therapeutic usefulness of IPs [106,107,108]. The ability of chalcogenylated-IPs to enhance antineoplastic effects at less toxic and equally effective doses while causing DNA, and cell death has been linked to an essential method in drug discovery as innovative chemotherapeutic drugs [109].

Other authors have tried a different strategy with various compounds. An anthelminthic medication called ivermectin is used to treat a variety of parasites [110]. Its strong anti-cancer properties have just been discovered. By inhibiting the p21-activated kinase (PAK-1)/Akt axis and modifying P2X4/P2X7 signaling, it causes cytostatic autophagy and necrosis in breast cancer cells, respectively [111,112]. Through inhibition of the WNT-TCF pathway, ivermectin suppresses the growth of xenografts of various solid cancer types without causing evident adverse effects [113].

According to reports, ivermectin can cause breast cancer cells to die by a combination of necrotic and apoptotic processes [114]. The pan caspase inhibitor Z-VAD-fmk, however, completely reverses the effects of ivermectin, indicating that ivermectin causes caspase-dependent apoptosis, which results in CML cell death. Additionally, it causes leukemia and other blood cancers to undergo chloride-dependent membrane hyperpolarization and cell death [115].

When it comes to generating caspase-dependent apoptosis in primary CML CD34+ and the CML cell line K562, ivermectin works far better than normal bone marrow (NBM) CD34+ cells. The conventional CML tyrosine kinase inhibitors’ in vitro and in vivo efficacy is also increased by ivermectin. Ivermectin lowers mitochondrial respiration in K562 and CML CD34+ cells, and inhibits respiratory complex I activity mechanistically (Table 1).

Remarkably, research has shown that NBM CD34+ cells exhibit reduced levels of mitochondrial respiration in contrast to malignant CD34+ cells. Moreover, NBM CD34+ cells exhibit a comparable pattern of mitochondrial dysfunctions to CML CD34+ cells when exposed to ivermectin, while NBM CD34+ cells exhibit a markedly lower sensitivity to the drug than CML CD34+ cells. These imply that NBM CD34+ cells can withstand mitochondrial dysfunctions better than CML CD34+ cells. Every time it is compared to normal counterparts, ivermectin consistently causes larger amounts of OS and damage to CML.

NAC, an antioxidant, restores the effects of ivermectin, indicating that OS is the mechanism of action in CML. The basic data for repurposing ivermectin for the treatment of CML is provided by an experiment [116]. According to the findings, ivermectin prevents mitochondrial respiration by lowering complex I activity in the mitochondria, which damages CML cells and causes an energy crisis. A common indicator of oxidative DNA damage is 8-hydroxy-2′-deoxyguanosine (8-OHdG). It appears that ivermectin’s production of 8-OHdG results from OS, which is brought on by mitochondrial malfunction. This is further supported by the observation that, in comparison to CML CD34+ cells, NBM CD34+ cells exhibit lower levels of 8-OHdG due to their decreased sensitivity to mitochondrial malfunction. Ivermectin’s ability to induce OS and apoptosis is greatly inhibited by the antioxidant NAC, indicating that oxidative stress and mitochondrial dysfunction are necessary for ivermectin’s activity in CML [116]. Transient receptor potential (TRP) channels, which are non-selective cation-permeable receptors found in the mitochondria, endoplasmic reticulum, and plasma membrane, have been extensively studied in relation to their critical function in controlling membrane voltage and cation concentrations [117]. Furthermore, TRP dysregulations’ role in chemoresistance and cancer progression has been documented in a number of scholarly publications. One of the TRP channels that affects the fine regulation of the proliferative signaling/cell death pathways is TRP vanilloid 1 (TRPV1), a ligand-gated cation channel opens. Different cancer models have shown that TRPV1 activation contributes to the triggering of cell death [118,119,120,121]. In addition, TRPV1 activation promotes anti-proliferative and pro-apoptotic actions in primary lymphoblasts derived from T-acute lymphoblastic leukemia patients as well as the Jurkat cell line [122]. According to a study, transient receptor potential vanilloid 1 stimulation slows down the proliferation of chronic myeloid leukemia cells and encourages their death. Calcium influx, OS, ER stress, mitochondrial failure, and caspase activation were all brought on by its activation. Remarkably, it was discovered that N-oleoyl-dopamine and the common medication imatinib worked in concert [123].

## 3. Future Perspectives

### 3.1. Nanoparticles

Nanomedicines are made up of 20–200 nm-sized particles. The primary application of nanoparticles in tumor treatment involved facilitating the delivery of anti-neoplastic medications. In actuality, nanoparticles are attractive delivery systems for improving medication distribution because of their biocompatible and biodegradable nature. Nonetheless, through many mechanisms, nanoparticles enhance the therapeutic effects of hematological neoplasms [124].

Copper oxide nanoparticles, or CuO NPs, generate oxidative stress and reactive oxygen species (ROS), which damage DNA and enhance the expression of death receptors [125]. Zinc dioxide (ZnO) nanoparticles were found, by Premanathan et al., to prefer killing human leukemia cells over healthy peripheral blood mononuclear cells [126].

Iron oxide nanoparticles, or Fe_3_O_4_ NPs, have been shown by Ahamed et al. to have selective effects on cancer cell viability and death, with no discernible effect on normal cells [127].

CuO NPs’ cytotoxicity was investigated in normal (PBMCs) and CML (cell-line K562) cells. Using K562 cell viability, oxidative stress, and apoptosis as benchmarks, potential cytotoxic processes were investigated [128]. Additionally, RT-PCR analysis was used to measure the mRNA levels of the anti-apoptotic Bcl-2 gene and the apoptotic genes P53, Bax, Caspase 3, and P53. By selectively killing cancer cells in a dose-dependent manner, CuO NPs had a specific influence on cell viability without harming healthy cells.

CuO NPs’ dose-dependent cytotoxicity against K562 cells was demonstrated by the production of ROS. The confirmation of the apoptosis triggered by CuO NPs was achieved by double labeling with propidium iodide and acridine orange. CuO NP exposure increased the expression of the tumor suppressor gene P53, and an increase in the Bax/Bcl-2 ratio indicated that the mitochondria-mediated pathway was implicated in the apoptosis caused by CuO NPs (Table 2). The authors also noted that after a 24 h exposure, the expression of the Caspase 3 gene did not change [128]. These chemical changes shed light on how CuO NPs suppress the development, production of reactive oxygen species, and programmed cell death of CML cells.

### 3.2. Ultraviolet Radiation

The two main photoproducts of UV-induced DNA damage in humans are pyrimidine pyrimidone and 2,3-cyclobutane pyrimidine dimer, which are repaired by nucleotide excision [24,129]. On the other hand, UV-B can cause a number of other problems, including apoptosis, by stimulating the creation of ROS [130,131]. It was demonstrated that reduced mitochondrial potential was linked to UV-induced ROS generation [132]. As a result, the way that cells respond to UV damage may involve many of the same elements that are linked to CML cells’ imatinib resistance: nucleotide excision repair, which is the most effective system for repairing DNA and is essential for maintaining genomic stability, neutralizing reactive oxygen species, apoptosis, and mitochondrial function.

A study examined the levels of OS, DNA damage, programmed cell death, and the expression of genes linked to apoptosis in BCR-ABL1 cells that were both susceptible and resistant to imatinib [133]. Either the Y253H mutation in the BCR-ABL1 gene or exposure to escalating imatinib (AR) doses caused the resistance. When cells resistant to imatinib were exposed to UV irradiation at a dosage rate of 0.12 J/(m^2^·s), greater DNA damage was observed, as shown by the T4 pyrimidine dimers glycosylase and human glycosylase (hOGG1), which recognized oxidative changes to DNA bases. The resistant cells showed increased vulnerability to UV-induced programmed cell death. When exposed to UV light, the cells’ initial mitochondrial membrane potential was higher than that of imatinib-sensitive cells. A substantial reduction in the expression of the succinate dehydrogenase gene, which codes for a part of the mitochondrial respiratory chain complex II and is implicated in apoptosis sensing, was noted by the authors. A portion of AR cells were found to have the Y253H mutation, even though the precise mechanism underlying these cells’ resistance to imatinib is unknown [133]. In conclusion, differing responses to endogenous and exogenous DNA-damaging agents, such as DNA repair and apoptosis, may explain why imatinib-resistant cells exhibit varying degrees of genomic instability compared to their imatinib-sensitive counterparts.

### 3.3. RNA Interference

**Figure 3:**
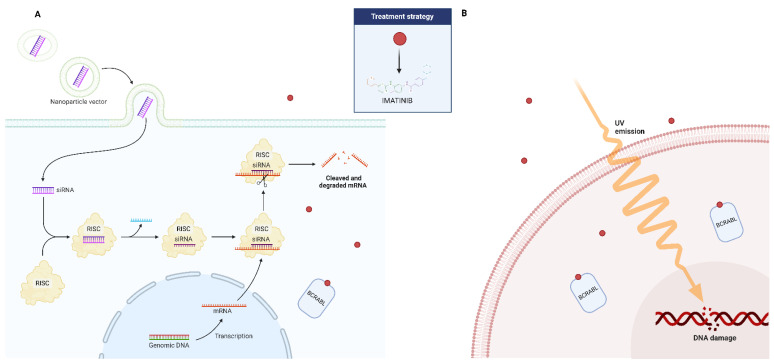
Possible adjuvant role of small interference RNA (siRNA) (**A**) and ultraviolet radiation (**B**) in the treatment of patients affected by CML who have developed resistance to imatinib.

The last several years have seen an incredible transformation in the treatment potential of many diseases due to RNA interference (RNAi)-based regulation of gene function. RNA interference (RNAi) is a biological defensive mechanism that has evolved over time. It is activated in response to double-stranded RNAs (dsRNAs), and works by selectively breaking down mRNA to silence genes. As a result, researchers have discovered that specific dsRNA sequences can be produced artificially to mute a gene responsible for a specific disease. Small interference RNA (siRNA) and micro-RNA (miRNA), two forms of dsRNA, are crucial elements that have been demonstrated to be utilized for gene silencing via the RNA interference mechanism [134]. Therapy for CML has also been tried with this strategy (Figure 3). In both CML the archetypal stem cell cancer and hematopoietic stem cell (HSC) self-renewal, the signal transducer and activator of transcription 5A (STAT5) plays a crucial role. In a study, the scientists established the involvement of this gene in CML disease and its persistence after targeted therapy using an RNA interference (RNAi)-based approach [135]. The findings demonstrated that STAT5A/STAT5B double knockdown inhibits the long-term clonogenic potential of both normal and CML HSCs and causes CML cell death. The pro-survival activity of STAT5A and STAT5B was comparable; however, the proliferation of normal and CML CD34+ cells isolated at diagnosis could not be inhibited by STAT5A attenuation alone. On the other hand, normal CD34+ and CML cells’ basal OS and DNA damage were sufficiently increased by STAT5A attenuation. Additionally, it increased the activation of the p53 (TRP53)/CHK-2 (CHEK2) stress pathway, impaired the ability to control external oxidative stress, and elevated the expression of prolyl hydroxylase domain (PHD)-3 (EGLN3) mRNA. These activities were particularly recovered only by STAT5A and its transactivation domain deficient mutant STAT5AD749. The proliferation of CML CD34+ cells from patients who had developed an imatinib resistance was likewise effectively inhibited by STAT5A attenuation [135]. These results show that STAT5A contributes to stress resistance in a specific way through non-traditional pathways, providing new avenues for eliminating the most primitive and TKI-resistant CML cells, as well as the possibility to eliminate persistent stem cell populations.

## 4. Conclusions

The main mechanism of action of chemotherapy is the induction of cell death via apoptosis; however, tumor cells may be resistant to this type of death [136]. As such, the search continues for new compounds capable of promoting multiple neoplastic cell death, with maximum efficacy and few adverse effects.

Furthermore, in the coming years, in addition to the role played by OS on apoptosis, other forms of cell death such as necroptosis, ferroptosis, and autophagy will need to be explored [137,138,139,140]. Managed necrotic pathways lead to a redox metabolome disruption, which depletes ATP and glutathione (GSH) and sets off an energy debacle.

An intriguing treatment target for CML is the interplay among redox metabolism, autophagy, and necroptosis. Autophagy is a cellular homeostasis system that functions to restore the cellular energy balance in response to oxidative and metabolic stress. It also helps cells survive under stressful conditions [141]. Furthermore, there is a crosstalk between autophagy and necrotic or non-apoptotic programmed cell death pathways, as well as apoptosis at the level of caspase-8 degradation [142].

According to Karvela et al., the activation of autophagy genes, such as ATG7, causes CML cells to exhibit increased rates of a basal autophagic flux [143]. Furthermore, it has been demonstrated that increased ROS and autophagy are directly related to treatment resistance in leukemia and CML development, respectively [144,145,146,147]. 

The chemical processes by which the tetrahydrobenzimidazole derivative TMQ0153 induced caspase-dependent apoptosis at low doses, along with a decrease in mitochondrial membrane potential (MMP) and an increase in cytosolic free Ca^2+^ levels, were finally studied by Song et al. [148]. It is interesting to note that TMQ0153 caused necroptotic cell death and ROS formation at higher concentrations, both of which may have been avoided with a pretreatment of N-acetyl-L-cysteine (NAC). The scientists noted higher ROS and lower ATP and GSH levels at necroptosis-inducing doses, along with the induction of protective autophagy. Bafilomycin A1 (baf-A1) and siRNA against beclin 1 are examples of inhibitors that inhibit autophagy, sensitize CML cells to TMQ0153, and increase necroptotic cell death. Crucially, TMQ153-induced necrosis resulted in the release of extracellular ATP, high mobility group box (HMGB1), and calreticulin (CRT) and ERp57 from the cell surface, indicating the compound’s ability to produce immunogenic cell death (ICD) markers. The in vivo reduction of K562 microtumor formation in zebrafish provided validation of TMQ0153’s anti-cancer potential. All together, these results show that in CML cell models, cellular stress, and redox manipulation by TMQ0153 concentration dependently result in several cell-death mechanisms, including controlled necrosis [148].

Although the exact role that OS plays in the pathogenesis of this entity is unknown, CML can be thought of as a typical model for the molecular pathogenesis of malignancy. The literature’s available data also present conflicting information. We believe that oxidative stress may have two distinct effects on the development of chronic myeloid leukemia: first, it may increase genomic instability and hasten the disease’s advancement to later stages linked to resistance to tyrosin kinase inhibitors; and second, it may facilitate the death of leukemic cells. It is noteworthy that distinct TKI generations seem to exhibit varying susceptibilities to oxidative stress, and their mechanisms of action vary significantly with respect to a given metabolic milieu. One suitable strategy to boost the effectiveness, lower resistance, and lessen the adverse effects of such medications may be to modulate oxidative stress.

However, to completely comprehend the actual mechanisms that can account for the cytophysiological processes that have been described, more research is necessary. It will actually be essential to determine if the oxidative stress caused by the drugs is the cause of these findings directly or indirectly. It should be also remembered that redox homeostasis disruptions are frequently only incidental or secondary.

In any case, a deeper understanding of the intricate mechanisms governing apoptotic processes and the consequences of oxidative stress will enable interventions to halt the progression of CML, thereby enhancing the efficacy of currently available treatments and potentially opening the door to more frequent and permanent CML interruptions—a goal that has become feasible in recent years. therapeutic CML intervention.

## Figures and Tables

**Table 1:** Chemical and natural substances alter the redox state and stimulating apoptosis of CML cells.

Compound	Mechanism	Reference
Curcumin	Causes alterations in the mitochondrial membrane potential	[72]
Betulinic acid	Influences the kinases gene expression and suppresses DNA topoisomerases	[81]
Fenretinide	Produces dihydroceramide that, in turn, leads to cytotoxic cell death	[87]
MRK-107	Causes a reduction in glutathione levels and an increase of lipid peroxidation	[97]
Ivermectin	Inhibits PAK/Akt axis and modifies P2X4/P2X7 signal, causing autophagy and necrosis	[103]

**Table 2:** The potential role of nanoparticles in causing DNA damage responsible for apoptosis of neoplastic cells from CML.

Structure	Target	Reference
Copper oxide (CuO) nanoparticles	Death receptors, causing DNA damage; suppressor gene P53, whose expression increases	[125]
Zinc oxide (ZnO) nanoparticles	Human leukemia cells	[126]
Iron oxide (Fe_3_O_4_) nanoparticles	Neoplastic cells, without effects on healthy ones	[127]

## Data Availability

Not applicable.
